# Effect of seasonal variation on hospital admission due to cardiovascular disease - findings from an observational study in a divisional hospital in Bangladesh

**DOI:** 10.1186/1471-2261-14-76

**Published:** 2014-06-13

**Authors:** Ranjit Chandra Khan, Debabrata Halder

**Affiliations:** 1 Department of Cardiology, Sher-e-Bangla Medical College, Barisal, Bangladesh

**Keywords:** Seasonal variation, Cardiovascular disease, Hospital admission, Bangladesh

## Abstract

**Background:**

Seasonal variation in the hospital admission due to cardiovascular disease (CVDs) has been widely reported. However, very limited data on Bangladesh is available regarding this matter. The aim of the current study was to investigate the effect of seasonal variation on hospital admission due to CVDs in a leading hospital of Bangladesh.

**Methods:**

Over a period of two years (from May 2010 to April 2012), the number of patients hospitalized due to various CVDs and number of death among these hospitalized patients were recorded on a day-to-day basis. The data were recorded according to the chief reason of hospital admission such as myocardial infarction or MI (acute, old and non-ST elevation), unstable angina (UA), exaggeration of stable angina, acute left ventricular failure (LVF), cardiomyopathy (ischemic and dilated) or heart failure, syncope and arrhythmia. The data were cumulated and analyzed on month-wise and season-wise manner.

**Results:**

A total of 8371 patients were admitted over the study period (5909 male and 2462 female; M/F ratio - 2.4:1). The highest number of patients were admitted during winter (n = 2839, 33.9%) and lowest during summer (n = 1648, 19.7%). The hospital admission was also significantly higher in winter compared to other seasons (p-value versus summer, autumn and spring was 0.018, 0.020 and 0.023 respectively). Acute MI (n = 2374), Acute LVF (n = 1582) and UA (n = 1277) were the top three reasons for hospitalization. Number of death also significantly higher in winter compared to other seasons (p-value versus summer, winter and spring was 0.044, 0.050 and 0.014 respectively).

**Conclusion:**

A seasonal variation in the hospital admission due to CVDs with a peak in winter was clearly demonstrated in the study. These data could be useful to improve causative prevention measures, therapeutic management, and educational strategies.

## Background

Cardiovascular diseases (CVDs) are the number one cause of death worldwide. An estimated 17.3 million people died from CVDs in 2008, representing 30% of all global deaths [1]. Several studies reported a seasonal variation in the hospitalization due to various CVDs with an increased rate during the winter [2-7].

Abrignani MG et. al. reported a seasonal variation in the number of admission in a western Sicily (Italy) hospital due to acute myocardial infarction (MI) [2] and angina [3]. They found a significant peak in winter. Loughnan ME et al. [4] also reported increased rate of hospitalization for acute MI in a hospital in Melbourne (Australia).

Indeed, Gotsman I et al. [5] observed the seasonal variation in hospitalization for heat failure (HF) in Heart Institute, Hadassah University Hospital, Jerusalem, Israel. They found a prominent seasonal variation with peak admissions during the winter. Some other authors from different countries also reported similar findings [6,7].

However, very limited data on Bangladesh is available regarding this matter. A better understanding of these seasonal patterns may provide novel avenues in cardiovascular prevention in Bangladesh. Considering the circumstances, we aimed to investigate the effect of seasonal variation on hospital admission due to CVDs in a leading hospital of Bangladesh.

The study was carried out in the Cardiology department of Sher-E-Bangla Medical College, Barisal. Barisal is one of the seven administrative divisions of Bangladesh, is located in the south-central part of the country and is having a population of 8,147,000 (according to Population Census 2011). This is the only medical college having cardiac care facilities in Barisal division.

## Methods

The number of patients hospitalized due to various CVDs and the number of death among these hospitalized patients from 1^st^ May 2010 to 30^th^ April 2012 were recorded in registry book on a day-to-day basis. The data were recorded according to the chief reason of hospital admission such as myocardial infarction or MI (acute, old and non-ST elevation), unstable angina (UA), exaggeration of stable angina, acute left ventricular failure (LVF), cardiomyopathy (ischemic and dilated) or heart failure, syncope and arrhythmia. The number of patients who died after hospitalization was recorded under ‘Cardiac Death’. The data were cumulated and analyzed on month-wise and season-wise manner.

Patients were diagnosed in accordance to standard practice in cardiology. For purposes of the study, the seasons are defined as follows: summer = May to July (average temperature 29.3°C); autumn = August to October (average temperature 25.1°C); winter = November to January (average temperature 19.8°C); spring = February to April (average temperature 27.4°C). Regional temperature was collected from the weather authority.

The adjusted seasonal average of patients admitted in cardiology department of the hospital was used as the primary study end point and was calculated by normalizing the total number of cases for each season to a standard 90-day long length. For example, the number of cases occurring in the winter equaled the number of cases from May 1^st^ to July 31^st^, 2010 plus cases occurring from May 1^st^ to July 31^st^, 2011 divided by the total number of days in these periods multiplied by 90. Adjusted seasonal mean values were reported for analysis. The same method was used by Spencer FA et al. [8] for reporting the seasonal distribution of acute myocardial infarction.

### Study ethics

The study protocol was reviewed and approved by the Sher-E-Bangla Medical College Ethical Committee (SBMC/Ethical Committee/23). Because the current study was performed as a retrospective study using the database and medical records, informed consent was waived by the committee.

### Statistical analysis

Statistical analyses were performed using SPSS 16.2.1 software package. To find out the significance in the differences of the primary end point between two seasons, t-tests were performed. p < 0.05 was considered statistically significant.

## Results

**Figure 1:**
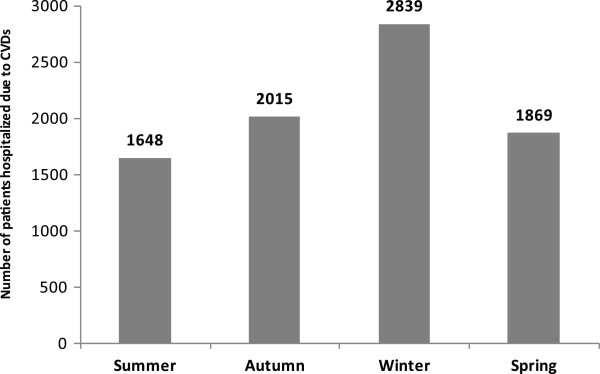
**Number of patients hospitalized due to CVDs reported each seasons in the Cardiology department of Sher-E-Bangla Medical College, Barisal, Bangladesh from May 2010 to April 2012.** Data reported as the adjusted seasonal average number of admission by seasons. The seasons are defined as Summer = May to July, Autumn = August to October, Winter = November to January, Spring = February to April; CVDs considered: myocardial infarction (acute, old and non-ST elevation), unstable angina, exaggeration of stable angina, acute left ventricular failure, cardiomyopathy (ischemic and dilated) or heart failure, syncope and arrhythmia.

**Figure 2:**
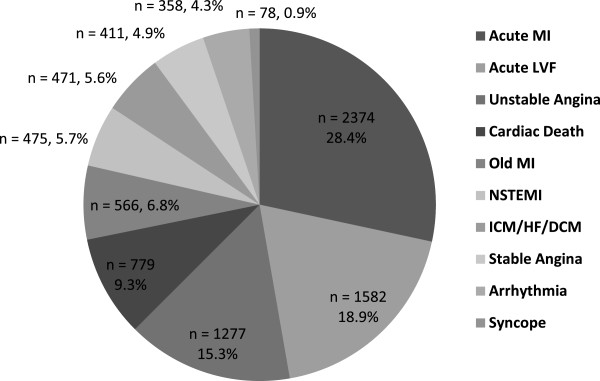
**Distribution of chief reasons of hospitalization in the Cardiology department of Sher-E-Bangla Medical College, Barisal, Bangladesh from May 2010 to April 2012.** Data reported as the adjusted seasonal average number of admission by diseases.

**Figure 3:**
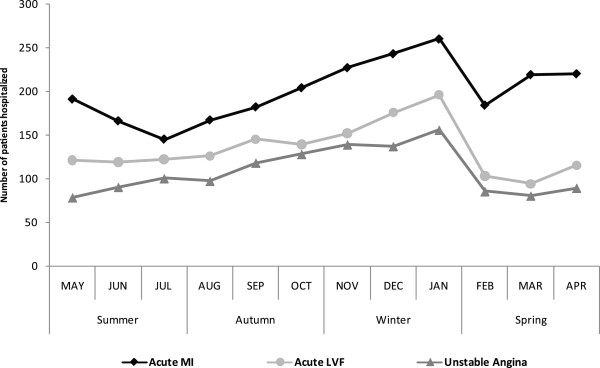
**Trend of hospital admission due to acute MI, acute LVF and unstable angina in the Cardiology department of Sher-E-Bangla Medical College, Barisal, Bangladesh from May 2010 to April 2012.** Data reported as month wise exact number of admission by three major cardiovascular diseases.

A total of 8371 patients were admitted over the study period (5909 male and 2462 female; M/F ratio - 2.4:1). The highest number of patients were admitted during winter (n = 2839, 33.9%) followed by autumn (n = 2015, 24.1%) and spring (n = 1869, 22.3%). The lowest number of patients were admitted during summer (n = 1648, 19.7%). The hospital admission was also significantly higher in winter compared to other seasons (p-value versus summer, autumn and spring was 0.018, 0.020 and 0.023 respectively). The results are summarized in Table 1 and number of hospitalized patients in these four seasons is graphically presented in Figure 1.Acute MI, acute LVF and UA were the top three reasons for hospitalization (Figure 2). 62.6% of the total patients were admitted due to these three reasons. In case of all these three reasons, a prominent peak in winter was observed (Figure 3) and number of hospital admission was higher in winter compared to other seasons.

**Table 1:** Cases of all cardiac incidences reported from May 2010 to April 2012 in the Cardiology department of a leading hospital in Bangladesh (Sher-E-Bangla Medical College)

	**Total Patients**	**Summer**	**Autumn**	**Winter**	**Spring**	**p – value (Winter versus other seasons)**		
	**no.**	**n (%)**	**n (%)**	**n (%)**	**n (%)**	**vs Summer**	**vs Autumn**	**vs Spring**
All Cases	8371	1648 (19.7)	2015 (24.1)	2839 (33.9)	1869 (22.3)	0.018	0.020	0.023
Acute MI	2374	491 (20.7)	541 (22.8)	714 (30.1)	628 (26.4)	0.012	0.015	0.082
Old MI	566	108 (19.1)	141 (24.9)	210 (37.1)	107 (18.9)	0.005	0.021	0.016
NSTEMI	475	65 (13.7)	125 (26.3)	191 (40.2)	94 (19.8)	0.002	0.009	0.002
Unstable Angina	1277	262 (20.5)	336 (26.3)	423 (33.1)	256 (20.1)	0.003	0.063	0.004
Stable Angina	411	39 (9.5)	66 (16.1)	192 (46.7)	114 (27.7)	0.039	0.037	0.126
Acute LVF	1582	354 (22.4)	401 (25.3)	513 (32.5)	314 (19.8)	0.050	0.079	0.017
ICM / HF / DCM	471	64 (13.5)	89 (18.9)	199 (42.3)	119 (25.3)	0.041	0.056	0.122
Syncope	78	9 (11.5)	18 (23.1)	34 (43.6)	17 (21.8)	0.001	0.003	0.012
Arrhythmia	358	80 (22.3)	106 (29.6)	119 (33.3)	53 (14.8)	0.052	0.485	0.059
Cardiac Death	779	176 (22.6)	192 (24.6)	244 (31.3)	167 (21.5)	0.044	0.050	0.014

Data reported as “n” refer to adjusted seasonal average number of admission by seasons and by diseases. p-values were calculated by t-tests. p < 0.05 was considered statistically significant.

### Acute MI

A total of 2374 patients were admitted due to acute MI which was 28.4% of the total number of hospital admissions. The highest number of patients were admitted during winter (n = 714, 30.1%) followed by spring (n = 628, 26.5%) and autumn (n = 541, 22.8%). The lowest number of patients were admitted during summer (n = 491, 20.7%). Except for spring, the hospital admission was significantly higher in winter compared to other seasons (p-value versus summer, autumn and spring was 0.012, 0.015 and 0.082 respectively).

### Acute LVF

A total of 1582 patients were admitted due to acute LVF which was 18.9% of the total number of hospital admissions. The highest number of patients were admitted during winter (n = 513, 32.4%) followed by autumn (n = 401, 25.3%) and summer (n = 354, 22.4%). The lowest number of patients were admitted during spring (n = 314, 19.8%). Except for autumn, the hospital admission was significantly higher in winter compared to other seasons (p-value versus summer, autumn and spring was 0.050, 0.079 and 0.017 respectively).

### Unstable angina

A total of 1277 patients were admitted due to UA which was 15.3% of the total number of hospital admissions. The highest number of patients were admitted during winter (n = 423, 33.1%) followed by autumn (n = 336, 26.3%) and summer (n = 262, 20.5%). The lowest number of patients were admitted during spring (n = 256, 20.0%). Except for autumn, the hospital admission was significantly higher in winter compared to other seasons (p-value versus summer, autumn and spring was 0.003, 0.063 and 0.004 respectively).

### Cardiac death

A total of 779 patients were died among the hospitalized patients. The highest number of patients were died during winter (n = 244, 31.3%) followed by autumn (n = 192, 24.6%) and summer (n = 176, 22.6%). The lowest number of patients were died during spring (n = 167, 21.4%). Number of death also significantly higher in winter compared to other seasons (p-value versus summer, autumn and spring was 0.044, 0.050, 0.014 respectively).

## Discussion

The present study showed a statistically significant seasonal variation in the hospital admission due to CVDs with a peak in winter in a leading hospital of Bangladesh. This is the first study in Bangladesh to observe such effects. However, the findings of the present study are similar with the results of several other studies [2-7] performed in different countries.

The cold weather and its relationship with the increased incidence of various CVDs leading to hospitalization have been explained by several authors [8-18]. In particular, the greater incidence of cardiovascular mortality and acute MI during the winter is well known [2,17-20]. The mechanisms involved here are multi-factorial. In cold conditions both increased sympathetic nervous activity and greater sodium intake lead to an increase in blood pressure, heart rate, and left ventricular end-diastolic pressure and volume [9-11] with, in turn, greater heart oxygen requirement and reduction of ischemic threshold [11] that may be clinically relevant in patients whose coronary circulation is already compromised. There may be also more dramatic events, such as sudden death, due to the increased frequency of cardiac arrhythmias, or, perhaps through rises in blood pressure, abrupt rupture of atherosclerotic plaques [11]. In cold weather, a greater tendency to clot in circulatory system has been demonstrated [12-15]. This could be related to plasma volume contraction (haemoconcentration) [13,15,16].

The greater incidence and severity of heart failure (HF) leading to hospitalization during the winter is also widely reported [6,7,21-23]. A patient with HF has little physiological reserve to deal with an increase in cardiac workload. Temperature reduction can cause physiological changes leading to HF decomposition and increased hospitalization rates (eg, overload secondary to increased heart rate and total peripheral resistance, changes of total extracellular volume secondary to decrease in water loss by transpiration and perspiration, increased blood pressure values, and arrhythmias) [7,21]. Moreover, higher rates of infectious diseases in winter, particularly respiratory tract infections, may play a role [24,25]. Again, C-reactive protein levels, a well recognized marker of the potential risk of cardiovascular events, shows a seasonal variation as well, characterized by a winter peak [26].

Blood pressure levels are higher during winter months [27]. When the temperature falls, a compensatory vasoconstrictive response, particularly to the skin, is observed. This is associated with an increased after-load for the failing heart, and is achieved by upregulation of the neurohumoral cascade and increased levels of vasoconstrictors. Therefore, cardiac work increases to overcome the rise in after-load, and at the end-stage the failing heart is unable to cope with this increased demand [28-30].

The present study has some limitations. First, it was a single-center-based observational analysis.

It may not reflect the complete scenario of Bangladesh. Second, the temperature of a specified geographic area may not accurately represent the actual individual temperature exposure, which is influenced by personal behaviors. Third, data on relative humidity was not included. Fourth, the influence of age was not investigated. Fifth, it included the number of death among the hospitalized patients and did not included out-of-hospital deaths.

## Conclusion

A seasonal variation in the hospital admission due to CVDs with a peak in winter was clearly demonstrated in the study. These data could be useful to improve causative prevention measures, therapeutic management, and educational strategies. For example, healthcare systems should adjust the availability of emergency services and other hospital resources to the most vulnerable periods. Susceptible patients should be informed of the increased risk during winter, and the demonstration of a higher-risk period could be useful for general practitioners to improve causative prevention measures, therapeutic management, and educational strategies.

## Competing interests

The authors declare that they have no competing interests.

## Authors’ contributions

RCK: study idealization and design, data validation, writing of the background, methods, results, discussion and conclusion. DH: data collection, statistical analysis and proof reading. Both authors read and approved the final manuscript.

## Authors’ information

Ranjit Chandra Khan: Principal author.

## Pre-publication history

The pre-publication history for this paper can be accessed here:

http://www.biomedcentral.com/1471-2261/14/76/prepub
